# Prime time for base editing in the mitochondria

**DOI:** 10.1038/s41392-022-01068-x

**Published:** 2022-07-06

**Authors:** Michael A. Morgan, Lucas Lange, Axel Schambach

**Affiliations:** 1 Institute of Experimental Hematology, Hannover Medical School, Hannover, 30625 Germany; 2 REBIRTH Research Center for Translational Regenerative Medicine, Hannover Medical School, Hannover, 30625 Germany; 3 Division of Hematology / Oncology, Boston Children’s Hospital, Harvard Medical School, Boston, MA 02115 USA

**Keywords:** Cell biology, Genetics, Preclinical research, Molecular engineering

A recent study published in *Cell* by the Kim lab represents a great advance towards expanding DNA editing possibilities to mitochondrial DNA (mtDNA) as they show reasonable targeted A-to-G conversions in human mtDNA using base editors to broaden the arsenal of available tools.^1^

**Fig. 1:**
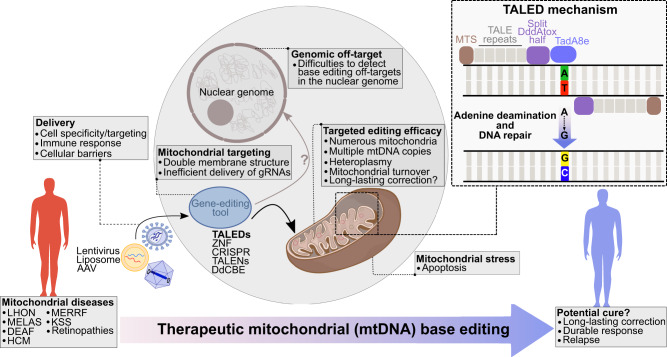
Challenges and promises of mitochondrial DNA (mtDNA) base editing applications to treat mitochondrial diseases. Efficiency of mtDNA base editing is dependent upon several factors, including efficient delivery to diseased cells, targeting of the mitochondria and mtDNA within these cells, as well as lack of off-target base editing activities. Abbreviations: LHON Leber hereditary optic neuropathy; MELAS mitochondrial encephalopathy, lactic acidosis and stroke-like episodes; DEAF deafness, sensorineural hearing loss; HCM hypertrophic cardiomyopathy; MERFF myoclonus epilepsy associated with ragged red fibers; KSS Kearns-Sayre syndrome; AAV adeno-associated virus; TALENs transcription activator-like effector nucleases; ZFN zinc-finger nucleases; CRISPR clustered regularly interspaced short palindromic repeats; TALEDs transcription activator-like effector deaminases; DdCBE DddA-derived cytosine base editor; MTS mitochondrial targeting sequence; DddAtox double-stranded DNA deaminase toxin A; TadA8e deoxyadenosine deaminase variant

Mutations in mitochondrial DNA (mtDNA) are linked to several diseases (Fig. 1), thus, development of gene therapy and editing approaches could lead to curative options for patients suffering from these illnesses. In this direction, gene editing tools such as zinc-finger nucleases (mitoZFN), transcription activator-like effector nucleases (mitoTALENs) and meganucleases (mitoARCUS) were shown to eliminate mutant mtDNA and shift heteroplasmy.^2–4^

Cho et al. generated A-to-G base editor constructs through a step-wise engineering approach in which three essential components were fused: (1) a mitochondrial targeting sequence (MTS), (2) a transcription-activator-like effector (TALE) protein designed to bind specific genes in the human mitochondria and (3) the TadA8e adenine deaminase (AD) variant known to catalyze adenine deamination in single-stranded DNA. Although the editing frequency in sequences proximal to the TALE-binding sites was only approximately 1% in human embryonic kidney (HEK) 293 T cells, this showed proof-of-principle for A-to-G conversions in double-stranded DNA in this context. The authors then focused on developing split TALE deaminases (sTALEDs) with increased efficiency and found that replacing uracil DNA glycosylase inhibitor (UGI) on one half of the sTALED by fusing TadA8e to DdCBEs that contain the cytosine deaminase DddA_tox_ resulted in A-to-G editing efficiencies of up to 19% and C-to-T conversion efficiencies of up to 14% in HEK 293 T cells.^1^ Complete removal of UGI further increased A-to-G editing efficiency to over 40%, while limiting C-to-T conversions.

Next, the authors modified TALED architecture to generate monomeric (mTALEDs) and dimeric (dTALEDs) adenosine base editors. Comparison of sTALEDs, mTALEDs and dTALEDs showed that sTALEDs had the highest overall A-to-G conversion efficiency upon analysis of twelve different sites within the mtDNA. Interestingly, mTALEDs and dTALEDs were more effective in some genes, offering additional possibilities to tailor mitochondrial gene editing.

The editing windows of these mt-DNA-directed TALEDs were typically between 10–20 bp, which is longer than those for nCas9 (D10A)-containing ABE, but was expected with the TALE-based approach. Of potential interest, editing patterns of the three different types of TALEDs were distinct, thus providing additional opportunities to fine-tune mtDNA genome editing. Important aspects such as cell viability, ratio of mtDNA to gDNA and oxidative phosphorylation levels were largely unchanged by the mtDNA editing procedure.

Whole mtDNA genome sequencing to assess off-target editing by the different TALED constructs revealed low-level off-target editing frequencies of 0.013–0.025% for sTALEDs, 0.009% for mTALEDs and 0.008% for dTALEDs. Although several potential off-target sites were identified in the nuclear genome, no A-to-G edits were detected by high-throughput sequencing at any of these sites following application of ten different sTALED designs, which may indicate that the MTS precludes localization of these TALEDs to the nucleus. Still, as the nuclear genome is far more complex than the 16.5 kb mtDNA genome, thus making detection of off-target base editing changes more difficult, further investigations such as unbiased genome-wide analyses are warranted to preclude and/or characterize these unwanted events. Proteome effects due to potential RNA editing events were not reported.

Another important aspect for clinical translation of gene editing strategies is delivery. Here, the mTALED design is advantageous as the coding sequence is approximately 3.1–3.5 kb, while the sTALEDs and dTALEDs are more than 5.3 kb (similar to DdCBEs). A recent study showed the feasibility of in vivo mtDNA base editing in mouse hearts.^5^ Using a cardio-tropic adeno-associated virus (AAV) serotype (AAV9.45) to deliver a DddA-derived cytosine base editor (DdCBE) to target the mouse mitochondrial *Nd3* gene, the authors showed targeted editing of 10–20% in the cardiac tissue of mice analyzed 24-weeks after tail-vein injection of 1 × 10^12^ AAV genomes per monomer per 8-week old mouse.^5^ The mtDNA editing efficiency increased to 20–30% upon application of the same doses to neonatal mice via temporal vein injection. Thus, it will be interesting to explore editing efficiency upon in vivo delivery of mTALEDs, which are small enough to be encoded into the approximately 4.5 kb genetic cargo space of AAV vectors. This is expected to be useful to show proof-of-concept, including efficacy and safety, for in vivo correction of mtDNA mutations, but also to generate more relevant disease models that may expedite our understanding of currently unknown pathogenic mechanisms.

As with any new technologies, questions regarding robustness of the approach always arise. In the case of mtDNA editing, several challenges are apparent. The double membrane structure of mitochondria has hampered CRISPR-Cas9 approaches to mtDNA editing due to inefficient transfer of the required guide RNAs into mitochondria. Cho et al. circumvent this with their TALE-based strategy, but could this also be resolved by an RNP-based approach with Cas9 in which the nuclear localization signal is exchanged for a MTS and the guide RNA is tethered to Cas9? Such strategies may simplify design of mtDNA editing approaches. Also, given that most human cells contain many mitochondria (up to hundreds) and that every mitochondria has multiple mtDNA copies (2–10), as well as the high mitochondrial turnover in cells, how often would such approaches need to be applied in order to achieve long-lasting robust correction? The cell line studies presented by Cho et al. suggest the possibility to select for edited mtDNA, at least in the case where they introduced *RNR2* gene mutations that conferred chloramphenicol resistance to cells. This approach showed that edited mitochondria could be retained and that isolated clonal populations had several A-to-G edits in the expected sites with up to 99.9% frequencies. Mouse experiments by others also provide in vivo evidence of long-lasting mtDNA editing.^4,5^ In summary, this exciting work is an important step towards extension of prime editing to mtDNA to allow all 12 possible base-to-base changes so that this technology can be used to develop more relevant disease models, increase our abilities to control cell functions and hopefully offer patients suffering with debilitating mitochondrial defects improved treatment options (Fig. 1).
